# Value of endometrial thickness for the detection of endometrial cancer and atypical hyperplasia in asymptomatic postmenopausal women

**DOI:** 10.1186/s12905-022-02089-y

**Published:** 2022-12-12

**Authors:** Linna Zhang, Ying Guo, Guxia Qian, Tao Su, Hong Xu

**Affiliations:** 1 The International Peace Maternity and Child Health Hospital, School of Medicine, Shanghai Jiao Tong University, Shanghai, 200030 China; 2 Shanghai Key Laboratory of Embryo Original Diseases, Shanghai, 200030 China; 3 Shanghai Municipal Key Clinical Speciality, Shanghai, 200030 China; 4 Putuo District Maternity and Child Care Center, Shanghai, 200062 China

**Keywords:** Asymptomatic, Endometrial carcinoma, Postmenopausal, Ultrasonography

## Abstract

**Background:**

The role of transvaginal sonography (TVS) in screening endometrial cancer and hyperplasia is significant in postmenopausal women. The objective of this study is to determine the endometrium thickness (ET) cut-off to distinguish premalignancy and malignancy in asymptomatic postmenopausal women.

**Methods:**

We retrospectively evaluated data of 968 eligible patients among 2537 asymptomatic postmenopausal women with ET ≥ 5 mm examined by TVS who were subjected to hysteroscopy and endometrial biopsy between January 1, 2017, and June 30, 2020 in an urban tertiary specialized hospital in China. The patients were divided into two groups according to the pathology outcomes: benign, and atypical hyperplasia (AH) and endometrial carcinoma (EC). The risk factors and the optimal cut-off of ET for detecting AH and EC were determined by logistic regression analysis and receiver operating characteristic curve.

**Results:**

2537 patients were offered hysteroscopy during a 42-month period. Finally, 968 patients were included for further analysis. Of these, 8 (0.8%) women were diagnosed with EC and 5 (0.5%) women with AH. The mean ET of AH and EC group was substantially higher than that in benign group (10.4 mm *vs.* 7.7 mm, *P* < 0.05). ET was significantly correlated with AH and EC shown by logistic regression analysis with an odds ratio (OR) of 1.252 (95% confidence interval [CI] 1.107–1.416, *P* < 0.001). The optimal cut-off value for AH and EC was found to be 8 mm with the maximum AUC of 0.715 (95% CI 0.686–0.743, *P* < 0.001), with a sensitivity of 0.846, a specificity of 0.609, positive likelihood ratio (LR+) of 2.164 and negative likelihood ratio (LR−) of 0.253.

**Conclusion:**

An ET cut-off of ≥ 8 mm shows a reasonable performance to detect AH and EC in asymptomatic postmenopausal women, thereby avoiding more invasive endometrial biopsy.

## Introduction

Endometrial carcinoma (EC) is the most common gynecologic cancer in the developed countries with steadily increasing incidence [1, 2]. Although 90% cases with EC present with postmenopausal bleeding [3], 15% cases of EC occur in women without vaginal bleeding [4]. The EC or atypical hyperplasia (AH) rate was reported to be 0.62% to 0.59% among asymptomatic postmenopausal women [5]. Given the fact that the five-year survival for patients with localized EC is 90%, detection of EC is quite pivotal for patients at early stage. American College of Obsetricians and Gynecologists (ACOG) guidelines support the transvaginal sonography (TVS) endometrial threshold of more than 4 mm to screen EC in women with postmenopausal bleeding [6].

TVS is performed in postmenopausal women due to a variety of indications such as pelvic pain, pelvic mass, and physical examination [7, 8]. However, it remains controversial that how to manage an incidental finding of thickened endometrium in asymptomatic postmenopausal women [3, 4, 9–11], consequently leading to a large number of invasive biopsies. Using an endometrial thickness (ET) cut-off value ≥ 4 mm, a prospective study indicated that only 3% of hysteroscopies were useful in the diagnosis of endometrial pre-malignant or malignant lesions [11]. In addition, the hysteroscopy and endometrial sampling could result in surgical risks including uterine perforation and bowel injury, patient anxiety as well as significant healthcare costs [3, 8, 12–14]. A previous study revealed 80% of the women undergoing hysteroscopy had a moderate to severe anxiety state [13]. The average cost of hysteroscopy and dilatation and curettage (D&C) turned out to be $2614 in Australia [14]. Therefore, the ET cut-off for identifying AH and EC in asymptomatic postmenopausal women warrants to be standardized.

In the current study, we sought to review our present practice and determine the ET threshold as well as the risk of EC and AH in asymptomatic postmenopausal women with ET ≥ 5 mm.

## Methods

### Patients and study design

A total of 2537 patients were provided with inpatient hysteroscopy between January 1, 2017, and June 30, 2020 in International Peace Maternity and Child Health Hospital (IPMCH), an urban tertiary specialized hospital in Shanghai, China. All medical records of these patients were reviewed. Patients who were eligible for both the inclusion criteria and exclusion criteria were included for the study.

We enrolled patients with the following inclusion criteria: (1) amenorrhoea for at least 12 months; (2) the indication of hysteroscopy was ET ≥ 5 mm which was incidentally found by TVS; (3) the ET must be evaluated by TVS in the same hospital, namely IPMCH; (4) patients had no obvious adnexal mass to eliminate the impact of hormone-secreting tumors on the endometrium; and (5) patients had no vaginal bleeding, no vaginal discharge, and no pelvic pain.

Exclusion criteria was as follows: (1) breast cancer patients with unknown information of tamoxifen treatment by reviewing the medical records were excluded; (2) patients with uterine malformation and those who were diagnosed with more than one malignancy were not included.

**Fig. 1:**
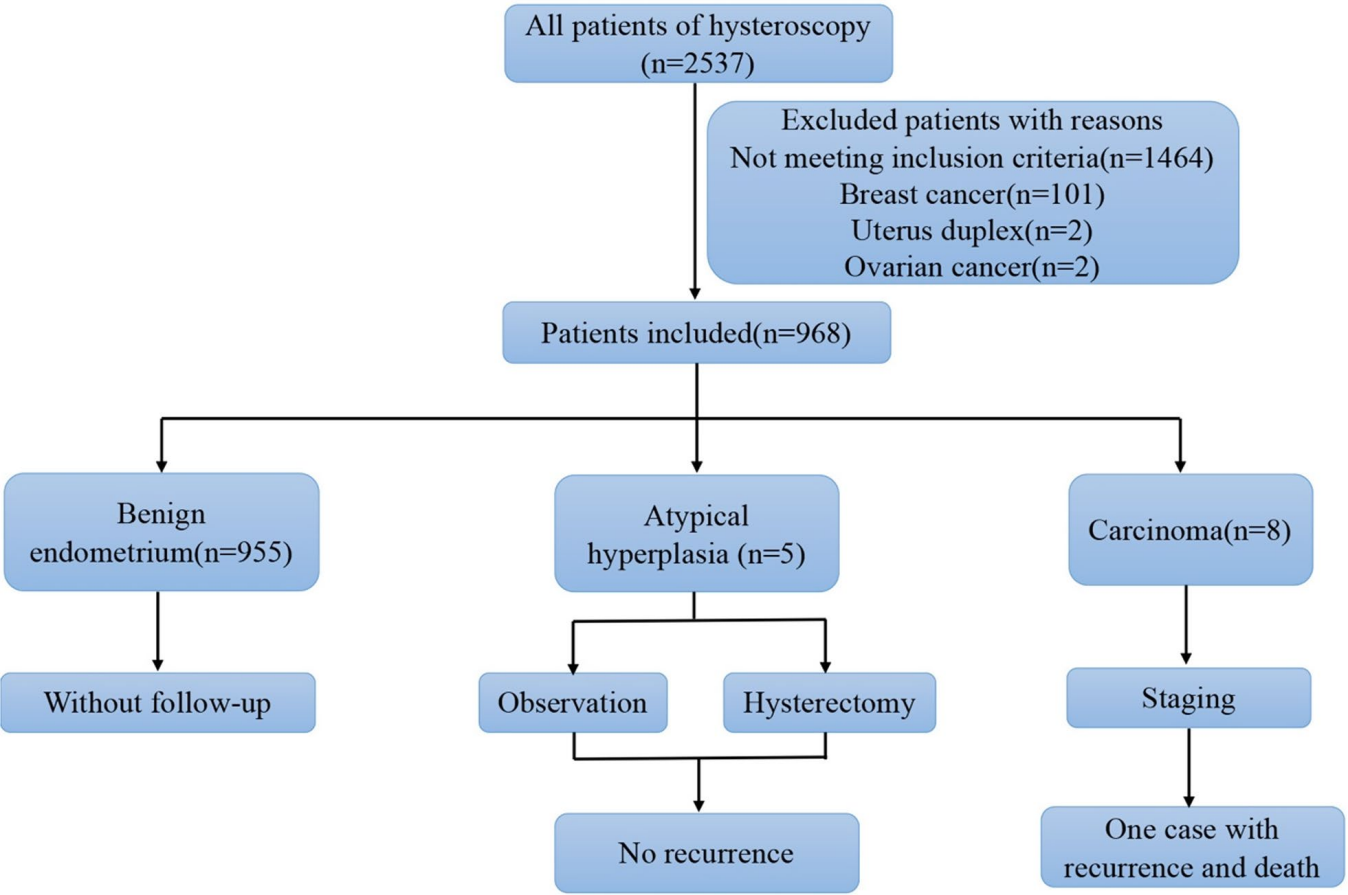
Flow diagram of the study

Among the 2537 patients to whom the hysteroscopy was offered during the period, 1464 patients having symptoms were excluded. 101 patients with breast cancer were excluded for the absence of information of tamoxifen therapy in the medical records. Patients diagnosed with uterus duplex (n = 2) and ovarian cancer (n = 2) were not included. Finally, 968 patients were available for further analysis (Fig. 1).

The endometrium of all these women was investigated by TVS in our hospital by two certified radiologists for routine physical examination using a transvaginal probe (GE Voluson scanner, E8, GE-Healthcare, Waukesha, WI, USA). The double wall ET was measured in the longitudinal plane at its thickest point. Hysteroscopies were conducted under total intravenous anesthesia. All the hysteroscopist were experienced with appropriate skill set. Endometrial biopsies were immediately immersed in 10% formaldehyde and sent to a pathology laboratory. The histopathological results were estimated by two pathologists.

Data were collected for all the eligible patients including age, body mass index (BMI), gravidity, parity, hypertension, diabetes, the image findings, histopathology results and outcome of all patients. All the 13 patients with premalignancy and malignancy were followed up until July 2022. All the procedures performed in studies involving human participants were in accordance with the ethical standards of Ethics Committee of IPMCH in Shanghai and with the 1964 Helsinki declaration and its later amendments or comparable ethical standards. The publication of this study has the permission of the patients.

### Statistical analysis

Continuous and nonparametric variables were analyzed using *t*-test and chi-squared test respectively. Logistic regression analysis was performed to determine risk factors for AH and EC. The optimal cut-off value of ET for AH and EC was determined by receiver operating characteristic (ROC) curve analysis by area under curve (AUC). Statistical analysis were performed with SPSS version 23.0 (IBM Corp., Armonk, NY, USA). A *P* value of < 0.05 was considered statistically significant.

## Results

TVS showed an ET ≥ 5 mm for all the included patients (n = 968). Pathologic outcomes of biopsy of the endometrium were listed in Table 1. Among the 955 patients with benign results, 696 (71.9%) were reported as endometrial polyps, 26 (2.7%) as hyperplasia, 32 (3.3%) as leiomyoma, 123 (12.7%) as atrophic endometrium, 27 (2.8%) as endometritis and 51 (5.3%) as normal endometrium. 5 (0.5%) cases had AH, and 8 (0.8%) were found to be EC.

**Table 1:** Pathologic outcomes in 968 menopausal asymptomatic postmenopausal women with a thickened endometrium ≥ 5 mm

Pathologic outcomes	n (%)
Benign endometrium	955 (98.7)
Endometrial polyps	696 (71.9)
Hyperplasia	26 (2.7)
Leiomyoma	32 (3.3)
Atrophic endometrium	123 (12.7)
Endometritis	27 (2.8)
Normal endometrium	51 (5.3)
Atypical hyperplasia	5 (0.5)
Endometrial carcinoma	8 (0.8)

Table 2 shows the demographic and clinical characteristics of patients. Patients were divided into 2 groups according to the pathologic outcomes (Group A, benign endometrium; Group B, AH and EC). The age of the patients varied from 46 to 87 years. The mean age in Group A was 61 years and in Group B it was 63 (*P* = 0.237). There was no significant difference between the BMI of the two groups (24.1 ± 3.37 *vs*. 24.2 ± 3.34, *P* = 0.928). Compared to Group A, it was comparable as with the other risk factors including gravity, parity, duration of menopause, hypertension, diabetes, history of cancer and family history of cancer in Group B. However, a significant difference was observed in the ET between Group A and Group B (7.7 ± 2.50 *vs*. 10.4 ± 4.50, *P* = 0.047). No complication was reported for all the included patients.

**Table 2:** Demographic and clinical characteristics of patients with different pathologic outcomes

	Group A (n = 955)	Group B (n = 13)	*P*
Age, yr, mean ± SD	61 ± 6.5	63 ± 6.4	0.237
BMI, kg/m^2^, mean ± SD	24.1 ± 3.37	24.2 ± 3.34	0.928
Gravity, mean ± SD	2 ± 1.2	3 ± 1.2	0.175
Parity, mean ± SD	1 ± 0.6	1 ± 0.6	0.805
Duration of menopause, yr, mean ± SD	9 ± 6.4	12 ± 9.0	0.222
Hypertension, n (%)	373 (39%)	4 (31%)	0.543
Diabetes, n (%)	76 (8%)	2 (15%)	0.643
History of cancer, n (%)	23 (2%)	1 (8%)	0.326
Family history of cancer, n (%)	17 (2%)	0 (0%)	1.000
Endometrial thickness, mm, mean ± SD	7.7 ± 2.50	10.4 ± 4.50	0.047

**Fig. 2:**
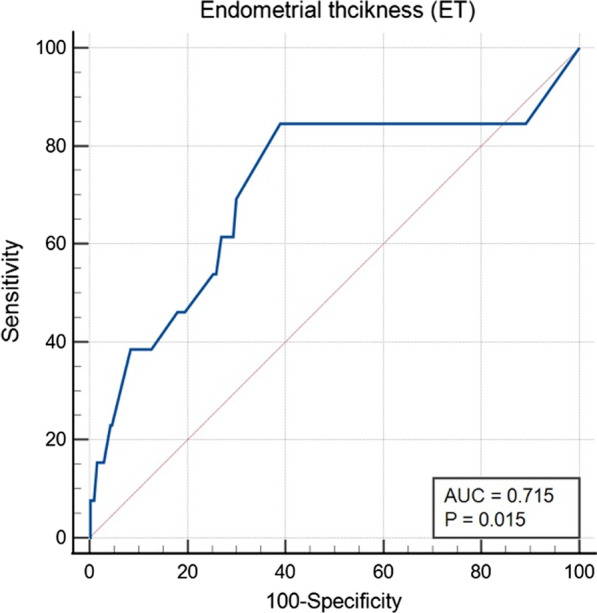
Effects of endometrial thickness detected using TVS in predicting the results of endometrial sampling

Logistic regression analysis showed that the ET was the risk factor for the AH and EC in asymptomatic postmenopausal women. The odds ratio (OR) for ET was 1.252 (95% confidence interval [CI] 1.107–1.416, *P* < 0.001). The diagnostic performance of ET was further analyzed by ROC curve analysis (Fig. 2). The optimal cut-off value for AH and EC was found to be 8 mm with the maximum AUC (0.715, 95% CI 0.686–0.743), with a sensitivity of 0.846, a specificity of 0.609, positive likelihood ratio (LR +) of 2.164 and negative likelihood ratio (LR−) of 0.253. The diagnostic value of ET with different cut-off value in endometrial lesions was compared in Table 3.

**Table 3:** Comparison of diagnostic value of endometrial thickness with different cut-off value in endometrial lesions

Cut-off (mm)	Sensitivity (% (95% CI))	Specificity (% (95% CI))	LR + (% (95% CI))	LR- (% (95% CI))
≧ 7.0	84.6 (57.8–95.7)	43.5 (40.3–46.6)	1.50 (0.97–1.79)	0.35 (0.09–0.44)
≧ 8.0	84.6 (57.8–95.7)	60.9 (57.8–64.0)	2.16 (1.37–2.66)	0.25 (0.07–0.44)
≧ 9.0	53.8 (29.1–76.8)	74.8 (71.9–77.4)	2.13 (1.05–3.40)	0.62 (0.30–0.92)
≧ 10.0	46.2 (23.2–70.9)	82.0 (79.4–84.3)	2.57 (1.13–4.52)	0.66 (0.35–1.08)
≧ 11.0	38.5 (17.7–64.5)	88.1 (85.9–90.0)	3.24 (1.26–6.45)	0.70 (0.39–1.28)
≧ 12.0	38.5 (17.7–64.5)	91.6 (89.7–93.2)	4.58 (1.72–9.49)	0.67 (0.38–1.28)

**Fig. 3:**
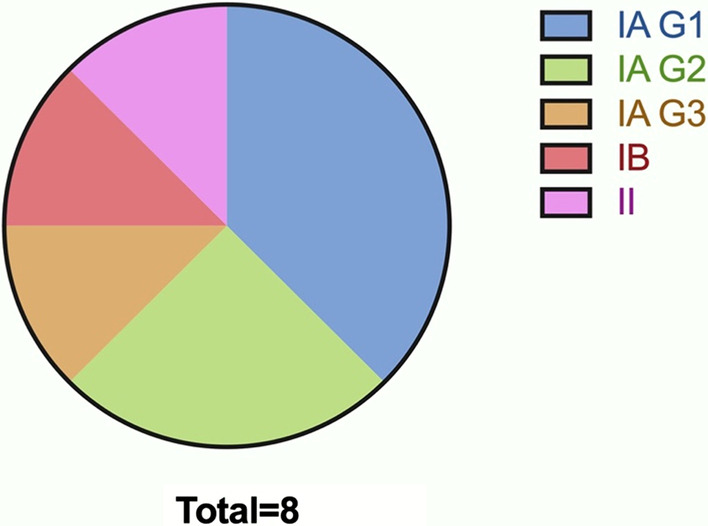
Distribution of pathologic outcomes among EC patients

Amongst women with the diagnosis of AH, 4 underwent hysterectomy, with one for observation. All 8 women with EC underwent staging surgery, with 3 at stage IA (grade 1, G1), 2 at stage IA (G2), 1 at stage IA (G3), 1 at stage IB and 1 at stage II (Fig. 3). 75% cases were found to be at stage IA. Follow-up information was available for all the 13 patients with AH and EC. With a follow-up period of between 2 and 5 years, one case of EC aging 70 years at stage IA (G2) exhibited recurrence and died 2 years after the staging surgery. The other 12 patients were alive without recurrence until July 2022.

## Discussion

The current retrospective study included 968 eligible cases and revealed that ET was the risk factor for the endometrial premalignancy and malignancy in asymptomatic postmenopausal women. The 8-mm ET was identified as the optimal threshold to detect AH and EC in asymptomatic postmenopausal women.

The strength of this study is that a large number of patients were included. But there are several limitations to our study. First, the analysis was limited due to the low prevalence of endometrial AH and EC as well as the retrospective property, which could result in bias. Second, it is a drawback that our analysis has not included the risk factors such as the use of hormone therapy and tamoxifen due to incomplete medical records, so the findings cannot be extrapolated to the whole population of asymptomatic postmenopausal women. Although it was revealed that there was no significant association between endometrial cancer and postmenopausal hormone usage [11, 15], the use of hormone therapy is necessary to be considered. Third, all the patients with benign conditions were not followed up. It remains unknown what effects the threshold would pose on the outcomes of these patients.

EC ranks fourth in female cancers, the management of which is of great concern. The surgical complications were reported especially in patients treated with laparotomy [15–17]. Given the fact that patients with late stage were more frequently subject to laparotomy, early identification of EC is quite important. The current study indicates that an incidentally-found ET of more than 8 mm in asymptomatic postmenopausal women might be recommended for further endometrial biopsy. Although different ET cut-off values had been investigated by various groups, there is no consensus as with the recommended ET to distinguish abnormal endometrium in postmenopausal woman without vaginal bleeding. The identified thresholds ranged 8–15 mm to distinguish EC or AH in asymptomatic women [3, 11, 18, 19]. Hysteroscopy was recommended to perform at a measurement of ET ≥ 8 mm in asymptomatic postmenopausal women in a study in Italy [11], while the threshold of 10 mm was suggested by several studies [3, 9, 18, 20]. The ET of 11 mm was identified as the optimal diagnostic threshold for atypical endometrium and malignancies [3, 4, 21, 22].Of note, the reported cut-offs were mostly based on AUC ROC method, which could be different from the ideal threshold when all the risk factors and patient outcomes are prospectively considered. Thus, prospective study with more samples will provide a more favorable threshold and diagnostic model for asymptomatic postmenopausal women in the future.

The present study also provides evidence for the fact that the incidence of malignancies or premalignancy is minimal in asymptomatic postmenopausal women. The prevalence of AH and/or EC was reported to be 0.62–9.9% [3, 5, 7, 10, 22–24]. As the endometrial condition is not routinely screened among asymptomatic women, the prevalence of EC and AH is difficult to estimate in this population. Thus, whether screening EC and AH is necessary warrants further investigation among postmenopausal women without symptoms. Many studies reported a significant association between EC and obesity [17, 25]. However, the benign and EC and AH groups showed no difference as with the BMI in our study, which may result from the small sample size in EC and AH group. Our study and previous studies consistently found that most cases were at stage I in postmenopausal women with no symptoms [3, 21, 26]. It was reported that there was no significant difference in rates of high-grade histology between asymptomatic women and women with abnormal uterine bleeding [26]. However, whether the survival rate benefits from the detection of EC in asymptomatic postmenopausal women compared to that in symptomatic postmenopausal women remains to be investigated. A further study including all the EC patients will provide more detailed information for pathological outcomes and survival rates.

This study has practice implications for clinicians to help provide with a threshold for management of asymptomatic postmenopausal women with endometrial thickening. However, the findings require confirmation in future clinical trials. The prospective study underlying the potential cut-off for endometrial premalignancy and malignancy desiderates to be further investigated and will shed light on the management of asymptomatic postmenopausal women with thickened endometrium.

## Conclusion

The mean ET of women with AH and EC is higher than that of patients with benign endometrium. An ET cut-off of ≥ 8 mm shows a reasonable performance to detect AH and EC in asymptomatic postmenopausal women, thereby avoiding more invasive endometrial biopsy.

## Data Availability

The data analyzed during the study is available from the corresponding author upon reasonable request.

## Abbreviations

EC: Endometrial carcinoma
AH: Atypical hyperplasia
ET: Endometrial thickness
TVS: Transvaginal sonography
AUC: Area under the curve
ROC: Receiver operating characteristic
OR: Odds ratio
CI: Confidence interval
LR+: Positive likelihood ratio
LR−: Negative likelihood ratio
BMI: Body mass index
